# The influence of a lost society, the Sadlermiut, on the environment in the Canadian Arctic

**DOI:** 10.1038/s41598-021-97631-7

**Published:** 2021-09-16

**Authors:** Finn A. Viehberg, Andrew S. Medeiros, Birgit Plessen, Xiaowa Wang, Derek Muir, Reinhard Pienitz

**Affiliations:** 1 Laboratoire de Paléoécologie Aquatique, Centre d’Études Nordiques, Pavillon Abitibi-Price, Université Laval, Québec, G1V 0A6 Canada; 2 Institut Für Geographie Und Geologie, University of Greifswald, Friedrich-Ludwig-Jahn Str. 16, 17487 Greifswald, Germany; 3 School for Resource and Environmental Studies, Dalhousie University, Halifax, B3H 4R2 Canada; 4 Helmholtz Centre Potsdam, GFZ German Research Centre for Geosciences, Climate Dynamics and Landscape Evolution, 14473 Potsdam, Germany; 5 Aquatic Contaminants Research Division, Environment and Climate Change Canada, Burlington, L7S 1A1 Canada

**Keywords:** Biogeography, Climate-change ecology, Freshwater ecology, Palaeoecology, Stable isotope analysis, Climate-change ecology, Freshwater ecology, Palaeoecology, Stable isotope analysis, Environmental chemistry, Environmental impact, Limnology, Climate-change adaptation, Climate-change impacts, Environmental impact, Sustainability, Environmental monitoring, Geochemistry, Climate change, Limnology, Palaeoclimate, Palaeontology, Sedimentology, Geochemistry

## Abstract

High latitude freshwater ecosystems are sentinels of human activity and environmental change. The lakes and ponds that characterize Arctic landscapes have a low resilience to buffer variability in climate, especially with increasing global anthropogenic stressors in recent decades. Here, we show that a small freshwater pond in proximity of the archaeological site “Native Point” on Southampton Island (Nunavut, Arctic Canada) is a highly sensitive environmental recorder. The sediment analyses allowed for pinpointing the first arrival of Sadlermiut culture at Native Point to ~ 1250 CE, followed by a dietary shift likely in response to the onset of cooling in the region ~ 1400 CE. The influence of the Sadlermiut on the environment persisted long after the last of their population perished in 1903. Presently, the pond remains a distorted ecosystem that has experienced fundamental shifts in the benthic invertebrate assemblages and accumulated anthropogenic metals in the sediment. Our multi-proxy paleolimnological investigation using geochemical and biological indicators emphasizes that direct and indirect anthropogenic impacts have long-term environmental implications on high latitude ecosystems.

## Introduction

Understanding the ‘push’ and ‘pull’ influence of environment on the migration and sustainability of peoples in northern North America over the last millennia is arguably one of the most important elements of understanding how recent climate change may affect society and lead to genetic adaptations^1,2^. The timing of migration has often been associated with paleo- temperature reconstructions that link evidence of distinctive material culture^3^ as well as the impact of subsistence practices in areas where hunting camps were established^4^ with shifting conditions. For the Dorset people, who were reliant on ice-dependent species such as walrus^5^, climate may have served as a “push” factor that served as a mechanism for northern migration during periods of time such as the Medieval Climate Anomaly (MCA). Conversely, the Thule were able to take advantage of increased activity of belugas and narwhals during longer open-water seasons, and migrations associated with the Thule expansion (circa 1250 CE) may have followed this transition until cooling associated with the Little Ice Age in the fifteenth century^3,6^. The Sadlermiut of Southampton Island (Nunavut, Arctic Canada) have often been referred to as descendants of the Dorset culture^7,8^ even though recent genetic evidence suggests they were a long isolated Thule population^9,10^. Archaeological evidence of stone-carved tools for walrus hunting, which is much more related to Dorset cultural practices than Thule^4,5^, is a prominent feature of winter hunting camps concentrated on the eastern side of Southampton Island in proximity to polynyas and ample walrus hunting grounds. Small, shallow ponds that are widespread in this area were used as staging grounds for the cleaning and preparation of subsistence harvest, and serve as sedimentary archives of the past presence and influence of the Sadlermiut, and their cultural practices, on the landscape.

High latitude freshwater ecosystems are often referred to as sentinels of environmental changes caused by climate variability and human activity^11^. Small and shallow lakes and ponds that characterize Arctic landscapes have a low resilience to buffer environmental change^12–14^, as well as catchment disturbances induced by prehistoric Inuit whalers^15^. Likewise, diffuse and point source disturbances can have disproportional effects due to the suboptimal environmental thresholds characteristic of biological communities of northern aquatic ecosystems^16^. Here, we show that a small subarctic pond in proximity of the archaeological site “Native Point” on Southampton Island evolved atypically after human activities initiated almost 800 years ago when Sadlermiut settled in the area. Our multi-proxy paleolimnological investigation uses geochemical and biological indicators to infer direct and indirect anthropogenic impacts. The lacustrine sediments collected from this site are highly sensitive environmental recorders that also allow us to pinpoint the first arrival of Sadlermiut culture, define their dietary shifts, and summarize the legacy of anthropogenic activities at “Native Point” since their first arrival.

### The legacy of the Sadlermiut on the environment

**Figure 1:**
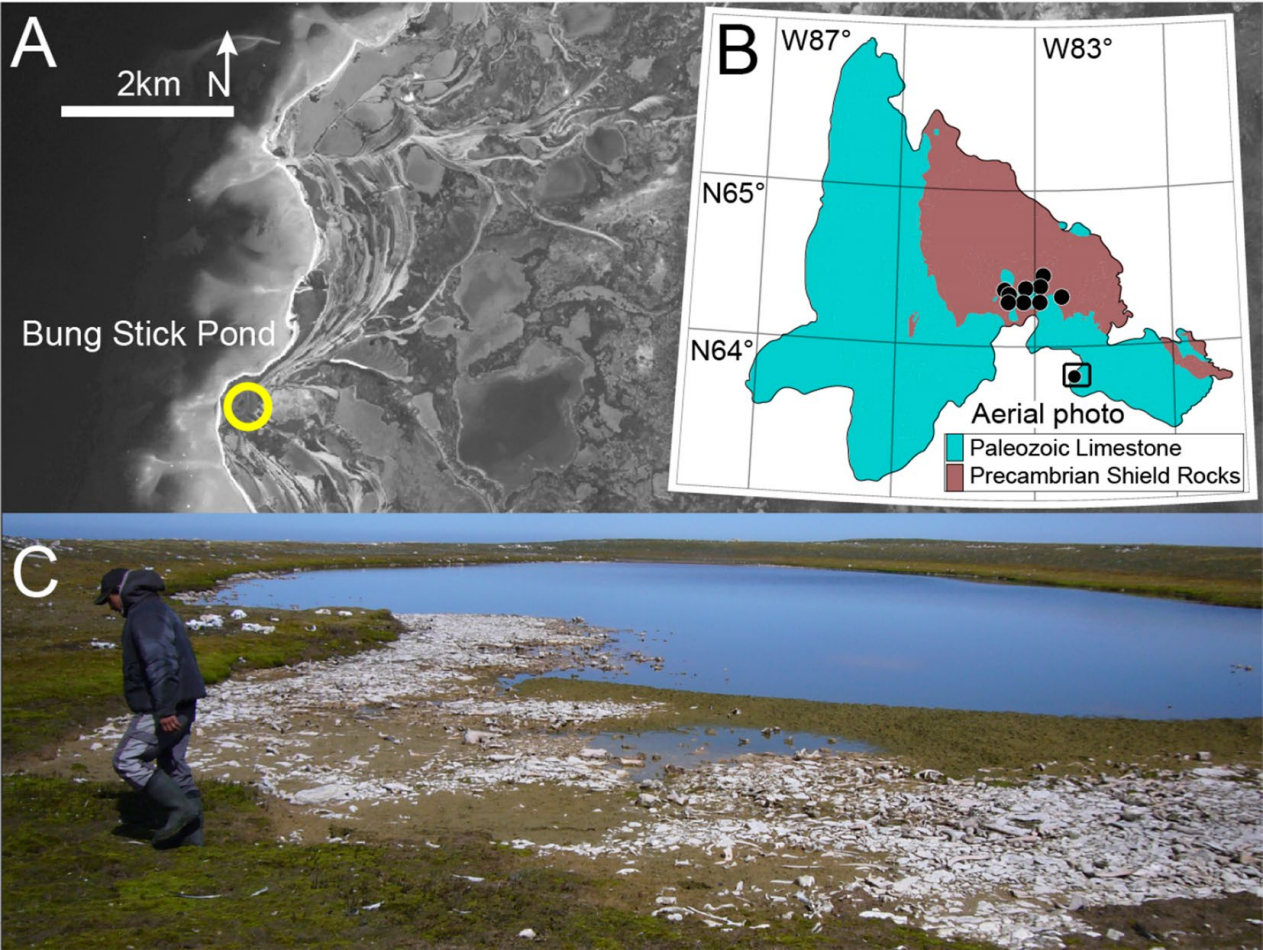
Bung Stick Pond and its catchment at Native Point, Southampton Island, Nunavut; (**A**) Aerial photo of Native Point (Orthoimage GéoBase, Natural Resources Canada), yellow circle—Bung Stick Pond; contains information licensed under the Open Government Licence—Canada; (**B**) Simplified geological map of Southampton Island^17^ and location of nine reference lakes and ponds; (Source: Geological Survey of Canada, "A" Series Map 1404A, 1977, 1 sheet, https://doi.org/10.4095/108900; contains information licensed under the Open Government Licence—Canada; georeferenced with Grass GIS 7.8.3; https://grass.osgeo.org/) (**C**) Photo of Bung Stick Pond facing northward, note scattered bones and antler fragments and partly paleozoic limestone gravel, informed consent for the publication of image has been obtained from Gabriel Bruce.

One of the richest archaeological sites found in the Canadian Arctic, the “Native Point” site was occupied by the Sadlermiut ca. 1250–1325 CE until decimated by disease introduced by European whalers in 1903^3–5^. The Sadlermiut village, referred to as the Tunermiut site^4^, consisted of numerous sod and winter houses that bordered a small shallow freshwater body (c. 20,000 m^2^), “Bung Stick Pond”. This site (Fig. 1A–C), and others in the well-known archaeological area of Native Point, offer a fascinating glimpse of an isolated society that evolved independently of modern-day Inuit and incorporated cultural elements of the Dorset peoples that vacated the area prior to the Thule migration^10^.

**Figure 2:**
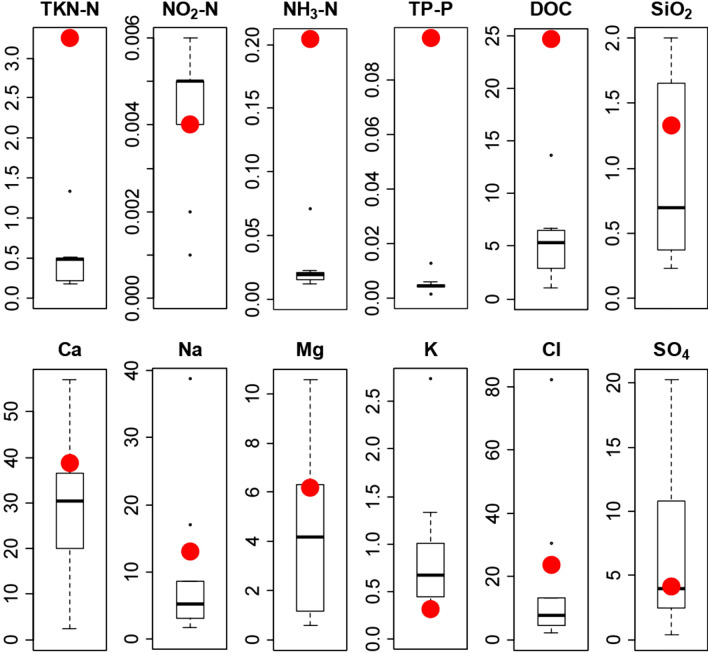
Box and whiskers diagram of water chemistry of nine lakes and ponds sampled on Southampton Island compared to Bung Stick Pond (red circle) (see Fig. 1). Nutrient indicators (top row) and major ion concentrations (bottom row) in mg L^−1^.

The heavy influence of Sadlermiut families processing food and leaving the remains of butchered carcasses to degrade in the pond is both visible and likely the main contributing factor for the difference in water chemistry that persists until today (Fig. 2). Southampton Island is characterized by a short vegetation period, ultra-oligotrophic freshwater ecosystems, and low sedimentation rates^18,19^. As such, the lakes and ponds of the area have low nutrient concentrations (i.e. total N and P; see Fig. 2), and the concentration of ions is dependent on soluble bedrock geology in their catchment, basin evolution since the last glaciation, distance to shore, and inputs from wildlife^14,18,20–22^. Here, the water chemistry of our study site, Bung Stick Pond, is an order or magnitude higher in concentrations of nutrients and organic carbon than in other lakes and ponds investigated on Southampton Island during the sampling period (Fig. 2). The only other eutrophic systems known in the region are those affected by waterfowl colonies^18^. Furthermore, the pond is characterized by an unusual high alkalinity caused by the catchment’s surface geology, which consists of Paleozoic limestone.

### The arrival and harvesting practices of the Sadlermiut

**Figure 3:**
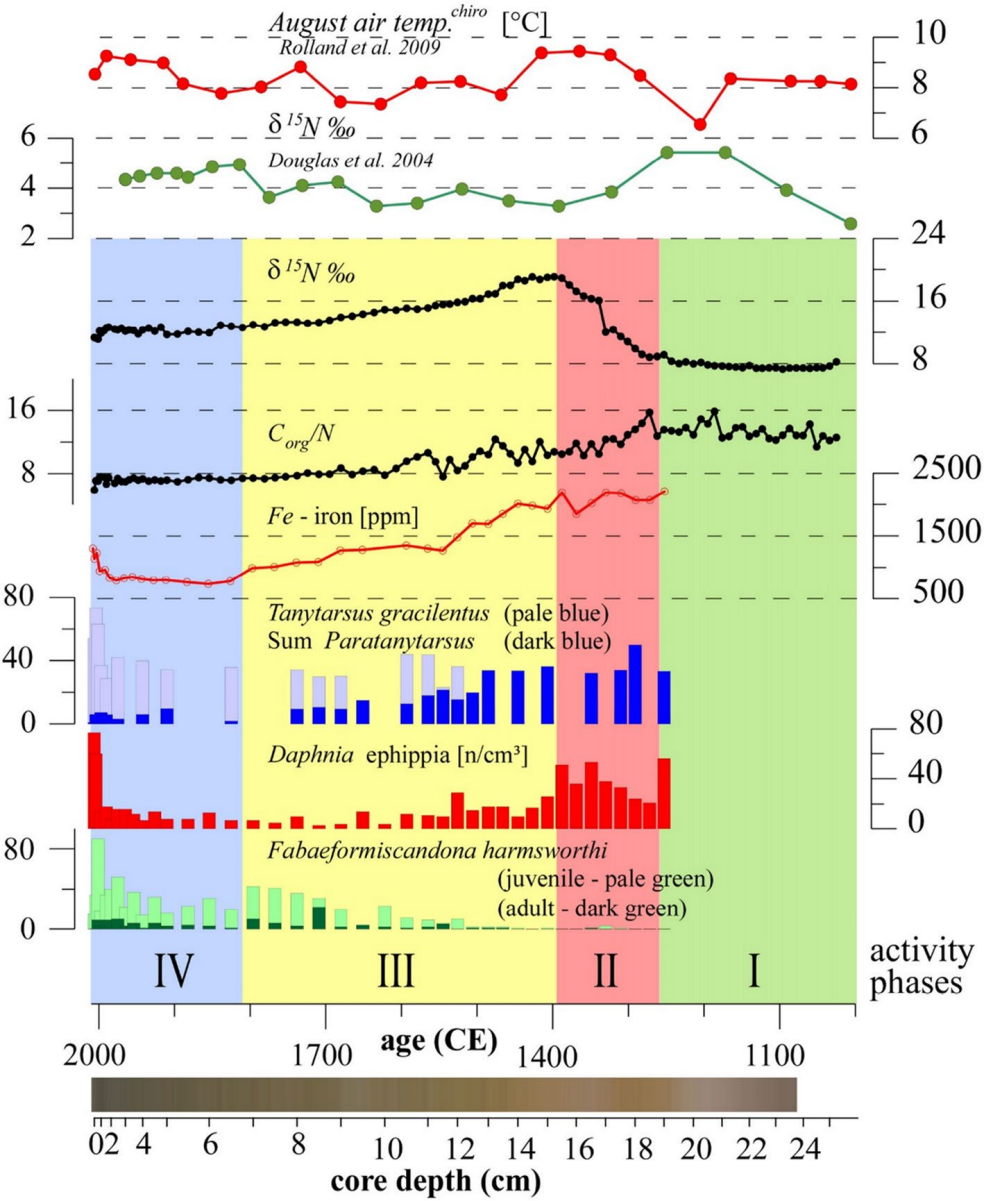
Nitrogen isotope analysis from paleo-Inuit harvesting sites and distinguishable phases at Bung Stick Pond cores. Inferred August air temperature based on chironomid remains from Southampton Island^19^. Earlier pronounced stable δ^15^N isotope record from sediment core tracingprehistoric Inuit whalers on Somerset Island^15^. Stable δ^15^N isotope record and TOC:TN-ratio from bulk sediment samples of core NP-3; iron (Fe) record from bulk sediment samples of core NP-2; selected relative abundance of chironomids of core NP-2, with *Tanytarsus gracilentus* (pale blue) and sum percentage of *Paratanytarsus* (dark blue); enumerated *Daphnia* ephippia (resting eggs) and *Fabaeformiscandona harmsworthi* (Ostracoda) valves of core NP-2 in individuals per cm^3^ with; adults (dark green), juveniles (pale green); interpreted activity phases I–IV at Native Point; sediment colors of age-corrected core NP-1.

**Figure 4:**
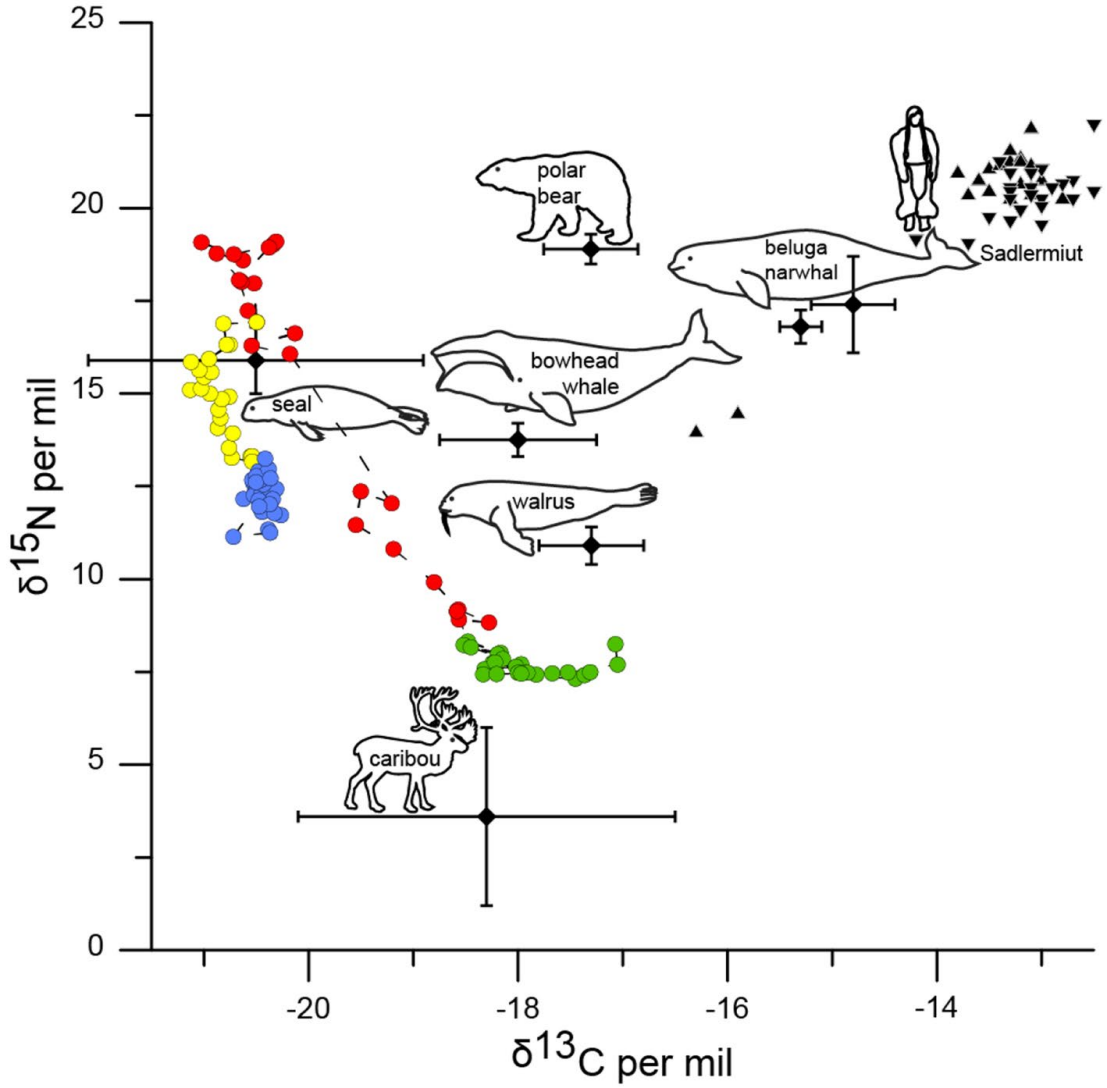
Relationship of δ^13^C and δ^15^N in organic material of sediment core NP-3 and bone collagen of the Sadlermiut and their potential diet. Circles indicate isotope excursion in organic material (sediment) in different time intervals; green (Phase 1): < 1253 CE, red (Phase 2): ~ 1250–1466 CE, yellow (Phase 3): 1466–1767 CE and blue (Phase 4): > 1767 CE; triangles show isotope data from human skeletal remains (bone collagen) in Sadlermiut burials from Coltrain (up)^23^, (down)^24,25^; whisker plots indicate modern range of isotope composition in muscle and blubber tissue of mammals supposedly included in the Sadlermiut diet from Hudson Bay or the Canadian Arctic/reports^26–30^.

The sediment history collected from Bung Stick Pond offers the possibility to track the aquatic system’s evolution since the arrival of the Sadlermiut when the site was used by the community for butchering of the collected harvest (Fig. 3). There is little archaeological evidence to suggest that the diet of Sadlermiut contained fish or any plants^4,5^, and the pond’s littoral zone is littered with skulls/skeletons at the bottom (see Fig. 1C). The predominant role of marine resources in Sadlermiut culture is also mirrored by the stable isotope signal in their adult bone collagen measured from burials^23–25^ (Fig. 4). Similarly, the surplus of organic material from the decaying process of carcasses in or around Bung Stick Pond carried the species specific isotope signal in the sediment. In general, heavier isotopes of nitrogen are enriched in predators relative to its food, which leads to high values in top predators of a food web^26–30^. Carbon isotope ratios usually show much less trophic enrichment, however a secondary fractionation process causes a positive offset in bone collagen in relation to soft tissue^26–30^ and apparently sediment samples.

The stratigraphic analysis of biological and geochemical indicators revealed four distinguishable phases that are attributable to the arrival and cultural practices of the Sadlermiut (Fig. 3). The reference condition of the pristine environment prior to Sadlermiut settlement (Phase 1; Fig. 3) is inferred by the low abundance of aquatic organisms (e.g., chironomids, cladocerans ephippia, ostracods) and δ^15^N values of around 8‰ at the base of the sediment core. During this time, the carbon:nitrogen ratio (TOC:TN) indicated mostly allochthonous inputs from the terrestrial environment^31^. An abrupt shift in geochemical indicators (Phase 2) suggests that the arrival of the Sadlermiut occurred between 1250 and 1300 CE. This period leads the earliest radiocarbon dated materials (1325 CE) found at the Sadlermiut heritage site^4^. Isotope analyses show a substantial increase in δ^15^N from about + 8 to + 19‰ (Fig. 3) and depletion of δ^13^C from about − 18 to − 21‰ (Fig.S2). Likewise, a decline in TOC:TN from 13 to 9 in bulk sediments indicates a large difference in the source of materials entering the lake and a sharp increase in aquatic production during this period^32^. Abnormally high iron concentrations were also observed starting from 1250 CE, potentially from blood washed into the system from butchered marine harvest.

The onset of Phase 3 (~ 1400 CE) suggests that settlement of the Sadlermiut camp supplied less external materials to the lake basin and a shift in the harvest of the Sadlermiut from a diet primarily comprised of marine mammals (e.g., seals, whales), which are characterized by the heavier δ^15^N and depleted δ^13^C (see Figs. 3 & S2), to one dominated by a more terrestrial origin (i.e., caribou). The shift in isotopic indicators, including the decrease of TOC:TN, during Phase 3 is concurrent with loss of macrophyte habitat as inferred from the chironomid data, notably the reduction of *Paratanytarsus* from 35 to < 10% relative abundance (Fig. 3)*.* At the same time, there was a substantial increase in the detritivore *Tanytarsus gracilentus* (from < 5 to 35%) (Fig. 3 & S4). This species is particularly interesting, as it has been associated with periods of algal decay^33^, likely representing increased productivity associated with the continuation of eutrophic conditions. The inferred human activity in the vicinity of the lake and subsequent eutrophication is in concert with the increase in abundance of resting eggs, ephippia, from planktonic *Daphnia* sp. in the corresponding sediment intervals^34^. The autochthonous population (i.e., juveniles > adults) of large ostracod species infer then a stabilized nutrient rich pond when *Fabaeformiscandona harmsworthi* became more abundant and a higher species richness is noted in ostracods (Fig. 3 & S5)^35^.

From ~ 1800 onwards, the biochemical indicators remain low and unchanged until 2006 (Phase 4), which suggests a steady decline of Sadlermiut activity at Native Point. Apparent increases of proxies from air-borne industrial pollution (e.g., tin, antimony, and lead) are characteristic for the top of the sediment record.

### The influence of climate on the Sadlermiut

The close relationship Inuit have with the environment offers a unique way of understanding the mechanisms behind a sustainable society under strict environmental controls. The colonization of the Hudson Bay region by Thule (1250–1400 CE) coincided with a period of warming during the Medieval Climate Anomaly^36^, which was also represented by a reduction of ice extent during this time^3^. Indeed, the projection of the regional paleo-temperature climate reconstruction^37^ onto the sediment history of Bung Stick Pond suggests that the onset of the Sadlermiut influence corresponded to a period of warmer conditions for the region. The intensification of the Little Ice Age (~ 1400–1700 CE)^19^ also coincided with the shift in dietary influence from seals and whales to more terrestrial-based mammals (i.e., caribou and muskox; Fig. 4), likely due to the need to obtain alternative food sources during periods of cooling and famine. Harsh winters and cooling in the Hudson Bay region during the nineteenth century took their toll on the population, and by 1896 there were only 70 Sadlermiut found^5,19^; documented encounters with the Sadlermiut were rare due to their isolation from contemporary Inuit communities and the lack of navigation maps for northern Hudson Bay by European whalers until the late nineteenth century^5^. Then, in the fall of 1902, a virulent gastrointestinal infection was brought to the community, which triggered its decimation over the winter of 1902–03^5^.

### A legacy of anthropogenic influence that outlasts the extinction of the Sadlermiut

**Figure 5:**
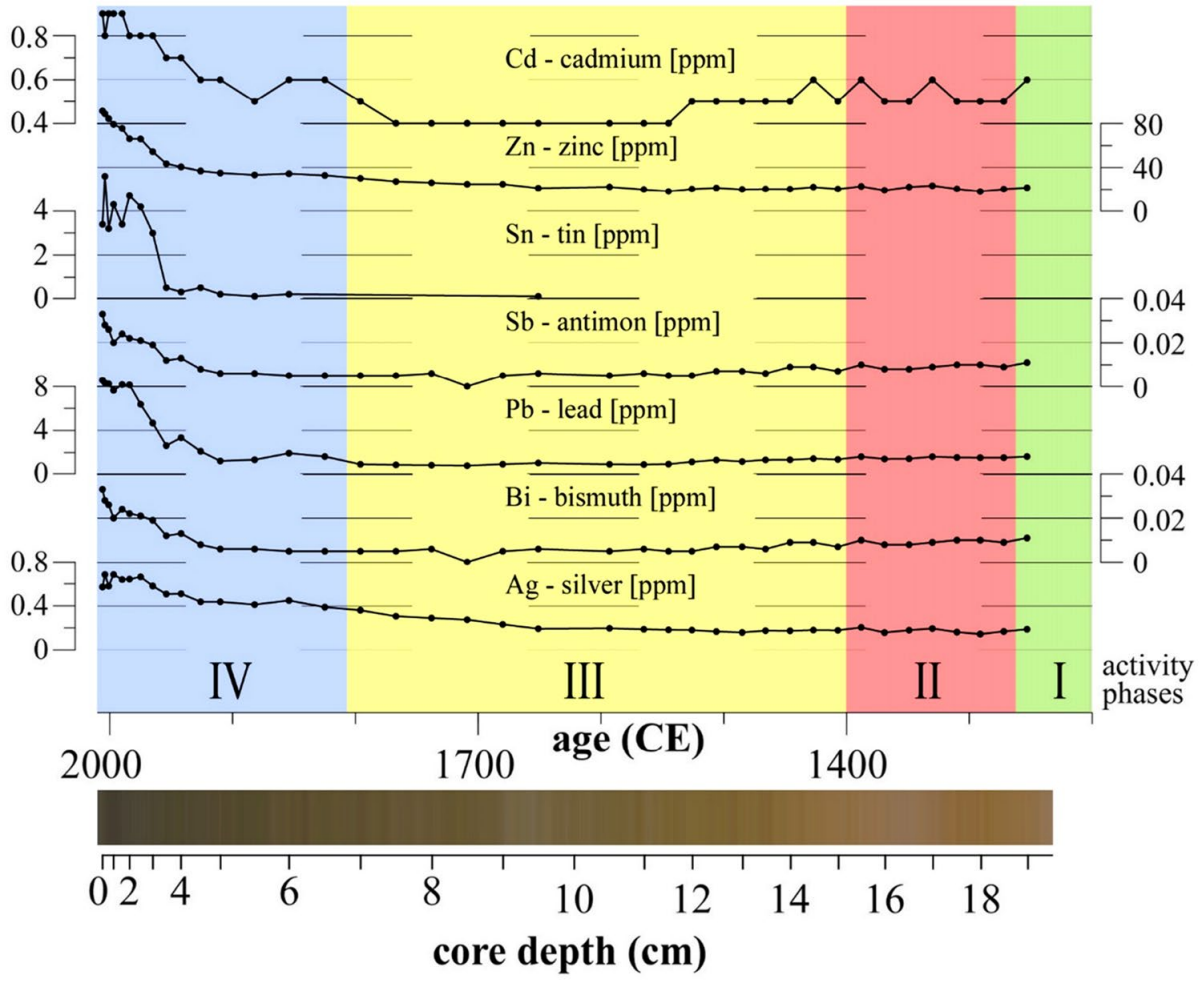
Metal concentrations of industrial air-borne pollution in sediment core NP-2; concentrations in ppm; interpreted activity phases I–IV at Native Point; sediment colors of age-corrected core NP-1.

Following the extinction and abandonment of Native Point, the nitrogen isotope data shows a slight decrease (Phase 4; Fig. 3)^37^. In contrast, analysis of metals from bulk sediments show enrichment with the start of the industrial era in the northern hemisphere^38^. A total of 47 elements were above detection limits in core NP-2 (Table S4). Trace elements Ag, Bi, Pb, Sb, Sn, and Zn all showed anthropogenic enrichment factors (EF; concentration post-1950/concentration pre-1800) of > 2 (Table S5). The sediment concentrations of each of the metals showed major increases from pre-industrial (~ 1850) to modern times consistent with industrial air-borne pollution (Fig. 5). Ag and Zn increased beginning ~ 1750–1800, while Bi, Pb, Sb and Sn showed increases occurring after 1900. The most striking EF was for tin (Sn), which had a rapid rise in concentrations from about 1900 (Fig. 5) and an EF of 72. Other trace elements including As, Cd, Cu, and Se showed modest enrichment (EFs 1.6–1.9) in post-1900 horizons (Table S5). So far, there is only one reference in subarctic Hudson Bay region that significant anthropogenic enrichment of Pb in post-1900 horizons (EFs 2–5×) has occurred^38^. Enrichment of metals is better known from ice cores from the Devon Ice cap (Devon Island Nunavut, Arctic Canada), which are in good agreement or show higher EFs than observations in the NP2 core. Noteworthy are anthropogenic enrichment of As and Bi^39^, Sb^40^, Pb^41^, Ag and Thallium (Tl)^42^, which originate from urban and industrial areas and linked to coal combustion and metal smelting. The overall comparison of ice cap ice cores and NP-2 EFs suggests that the inputs of Ag, Bi, Pb, Sb, and Ag are influenced by long-range transport from Eurasian sources^40,42^. Historical profiles are not available for Sn in Arctic sediment, peat, or ice core archives. Elsewhere, peat cores in the UK record deposition of Sn from regional tin mining and smelting^43^.

In concert with recent anthropogenic deposition of contaminants, an eutrophication trend can be inferred from more abundant remains of aquatic microfauna (i.e., chironomids, cladocerans, and ostracods) in the uppermost lake sediments (Fig. 3). Likewise, the sediments are composed of highly organic material (mean 15 wt%), which accumulates toward the core top exceeding 30 wt% (Fig. 3).

All these data indicate the extreme vulnerability and low resilience of small Arctic ponds as the effects of human activities at this site are still prevalent after more than 750 years. The sediment archive ipso facto records the influence of the Sadlermiut on the environment since their arrival and until the last of their population succumbed to disease in 1903. Furthermore, the continued contamination by airborne metal pollutants of remote Arctic landscapes since industrialisation is evident.

## Methods

### Field methods

Water temperature, pH, and specific conductivity of the Southampton Island’s waters were measured in the field with a multisensor Quanta Hydrolab water profiler. For chemical analyses, water samples were taken at a water depth of 50 cm and stored in conditioned PE-bottles stacked in a cooler box until samples were split and pre-treated in the field within 12 h according to the protocol of the Analytical Methods Manual of Environment Canada laboratories^44^ (data see Table S1).

### Coring and sampling technique

An Aquatic Research gravity corer with an internal tube diameter of 6.7 cm was used to collect three parallel sediment cores from an inflatable boat at N 63.7609°, W 82.5093° (WGS 1984). Core NP-1 (length 23.0 cm) was reserved for ^210^Po dating analyses. Core NP-2 (length 20.0 cm) was used for bioindicators and metal analyses. Core NP-3 was reserved for total organic carbon, total nitrogen, stable isotope analyses and radiocarbon samples (length 25.5 cm). The undisturbed core sediments were subsampled in the field and stored cool until freeze-dried.

### Geochronology

The sediments from core NP-1 were dated using alpha spectrometry of ^210^Po. Homogeneous freeze-dried portions of 33 samples including 1 set of replicates were treated using a variation on the Eakins and Morrison polonium (Po) distillation procedure, which assumes equilibrium between ^210^Po and ^210^Pb^45^. Unsupported ^210^Po showed a smooth exponential decline with depth and sedimentation rates and estimated dates were obtained through the Constant Rate of Supply (CRS) model^46^ (data see Table S2). For radiocarbon ages, degraded macro-remains from core NP-2 were submitted to *Cologne AMS* (COL) and *Keck Carbon Cycle AMS Facility* (UCIAMS) prepared following standard procedure for acid extraction^47^. The radiocarbon ages of all samples were calibrated into calendar years (CE) applying an INTCAL20 calibration curve^48^. Samples COL 4234 and UCIAMS-168600 were corrected for the local marine reservoir effect of ΔR = 263 ± 48 years following^49^ (data see Table S3). The sedimentation rates were modelled using Bayesian statistics^50^, based on nine levels of ^210^Po ages and three radiocarbon ages (Figure S1).

### Stable carbon and nitrogen isotopes

Sediment samples were freeze-dried, grounded, and analysed for their total organic carbon (TOC) and δ^13^C_org_, as well as total nitrogen (TN) and δ^15^N using an elemental analyzer (NC2500 Carlo Erba) coupled with a ConFlo III interface to a DELTAplusXL mass spectrometer (Thermo Fischer Scientific, Germany) at the GFZ, Germany. The reproducibility for replicate analyses is 0.2% for TOC and TN, and 0.2‰ for δ^13^Corg and δ^15^N.

### Metal analyses

Freeze dried sediments were analyzed for 47 elements using standard analytical protocols^44,51^ at the National Laboratory for Environmental Testing (Burlington, Ontario, Canada). Samples were digested with nitric/hydrochloric acid (1:3) on a hot block digestion system. The digests were diluted with water and analyzed by an inductively coupled argon plasma-collision/reaction cell mass spectrometer (CRC-ICP) using discrete sampling pneumatic nebulization. Percent recovery and precision of elemental analysis were based on standard sediment reference materials (NRC MESS-3, NIST RM 8704 and LKSD-3)^51^ (presented data see Table S4). Enrichment factors were calculated in relation to pre-industrial era (Table S5).

### Bioindicator analyses

Freeze-dried samples from core NP-2 were screened for remains of common bioindicators. Diatom and chrysophytes samples were prepared by using standard extraction techniques with chemical reagents H_2_O_2_ (30%) or sulfuric/nitric acids (50/50)^52^. Simple wet mounts without use of a chemical reagent also did not reveal any remains. For subfossil chironomid head capsules all species were isolated and mounted onto glass slides with Hydromatrix, using standard techniques^53^. Three intervals contained a low abundance of chironomids; 1.5 cm (17.5), 18 cm (10.5), and 19 cm (40.0). Specimens were identified using stereomicroscopes^54,55^. For ostracods and cladoceran ephippia, samples were soaked in a saturated Calgon solution and frozen to − 20 °C for 12 h^56^. Samples were then washed gently on a 125 µm screen with warm water and again freeze–dried. The complete material was screened under a dissecting microscope at 40 × magnification in a picking tray. All ostracod valves and cladoceran ephippia were isolated, enumerated, and identified^57–60^. Species composition clearly indicate a non-marine/freshwater environment throughout the core.

Informed consent for the publication of image has been obtained from Gabriel Bruce in Fig. 1c.

## Supplementary Information

Supplementary Information.
