# Supplementary material for: The influence of a lost society, the Sadlermiut, on the environment in the Canadian Arctic
Source: Sci Rep. 2021 Sep 16;11:18504. doi: 10.1038/s41598-021-97631-7 (PMC8446097; doi:10.1038/s41598-021-97631-7)
Supplement: Supplementary file 1 — Supplementary Information. [file 41598_2021_97631_MOESM1_ESM.docx]

**Supplementary Information for**

The influence of a lost society, the Sadlermiut, on the environment in the Canadian Arctic

Finn A. Viehberg, Andrew S. Medeiros, Birgit Plessen, Xiaowa Wang, Derek Muir, Reinhard Pienitz

Finn A Viehberg

Email: [finn.viehberg@uni-greifswald.de](mailto:xxxxx@xxxx.xxx)

**This PDF file includes:**

Supplementary text

Figures S1 to S5

Tables S1 to S5

SI References

Supplementary Information Text

**Water chemistry**

The field campaign to sample ponds and lakes on Southampton Island, Nunavut, Canada took place between 21.-24.07.2006 (Table S1). For chemical analyses, water samples were taken at a water depth of 50 cm and stored in conditioned 500 mL PE-bottles and stacked in a cooler box until samples were split and pre-treated in the field within 12 hours according to the protocol of the Analytical Methods Manual of Environment Canada laboratories [^1^](#_ENREF_1). The samples were then stored at 4 °C, shipped to, and analyzed by, the National Water Research Institute (Burlington, Ontario) for 10 parameters: chlorophyll-a (Chla), dissolved organic carbon (DOC), dissolved inorganic carbon (DIC), total Kjeldahl nitrogen (TKN), total phosphorus (TP), sulfate (SO_4_), chloride (Cl), silicate ions (SiO_2_), calcium (Ca), and sodium (Na).

**TOC, δ^13^C_org_ and TN, δ^15^N measurements**

Total organic carbon (TOC) and δ^13^C_org_, as well as total nitrogen (TN) and δ^15^N were determined using an elemental analyzer (NC2500 Carlo Erba) coupled with a ConFlo III interface to a DELTAplusXL mass spectrometer (Thermo Fischer Scientific, Germany) at the GFZ, Germany [^2^](#_ENREF_2).

The TOC content and δ^13^C_org_ values were determined on in-situ decalcified samples. About 3 mg of sample material were weighted into Ag-capsules, dropped first with 3 % and second with 20% HCl, heated for 3 h at 75°C, and finally wrapped and measured as described above. The calibration was performed using elemental (Urea) and certified isotope standards (USGS24, CH-7) and proofed with a soil reference sample (Boden3, HEKATECH). For TN and δ^15^N determination, around 10 mg of sample material were loaded into tin capsules. The nitrogen content was calibrated against Acetanilide, whereas for the nitrogen isotopic composition two ammonium sulphate standards (e.g. IAEA N-1 and N-2) were used. The reproducibility for replicate analyses is 0.2% for TOC and TN, and 0.2‰ for δ^13^C_org_ and δ^15^N. Results and mass ratio TOC/TN are plotted vs age model (Figure S2).

**Metal analyses**

*Sampling*

Core NP-2 (0.5 cm sections) was analyzed for 47 elements using standard analytical protocols [^1^](#_ENREF_1) at the National Laboratory for Environmental Testing (Burlington, Ontario, Canada). Further analytical methods followed previous paleolimnological studies [^3^](#_ENREF_3). Sediments were digested with nitric/hydrochloric acid (1:3) on a hot block digestion system. The digests were diluted with water and analysed by an inductively coupled argon plasma-collision/reaction cell mass spectrometer (CRC-ICP) using discrete sampling pneumatic nebulization. Percent recovery and precision of elemental analysis based on standard sediment reference materials (NRC MESS-3, NIST RM 8704 and LKSD-3) [^4^](#_ENREF_4). (See Figure S3 and Table S4&S5 for selected metal analyses).

*Results and Discussion*

Crustal elements Al, Fe, Ca, Mg, Mn, and Ti showed declining concentrations for horizons dated from 1100 CE to the mid-20th century (Figure S3), thus reflecting a decrease of erosional effects in the catchment paired with an increase in organic sedimentation rates as reflected in the age-depth model. Furthermore, three relatively abundant crustal or lithospheric elements Na, P, and K actually increased over time although most of the increase was in the 20th century (Figure S3). The sharp increases in K, Na, and P and the declines of Ca and Mg within the upper 1.0 cm may reflect greater exchanges with the water column, which is known for responsive and soluble elements.


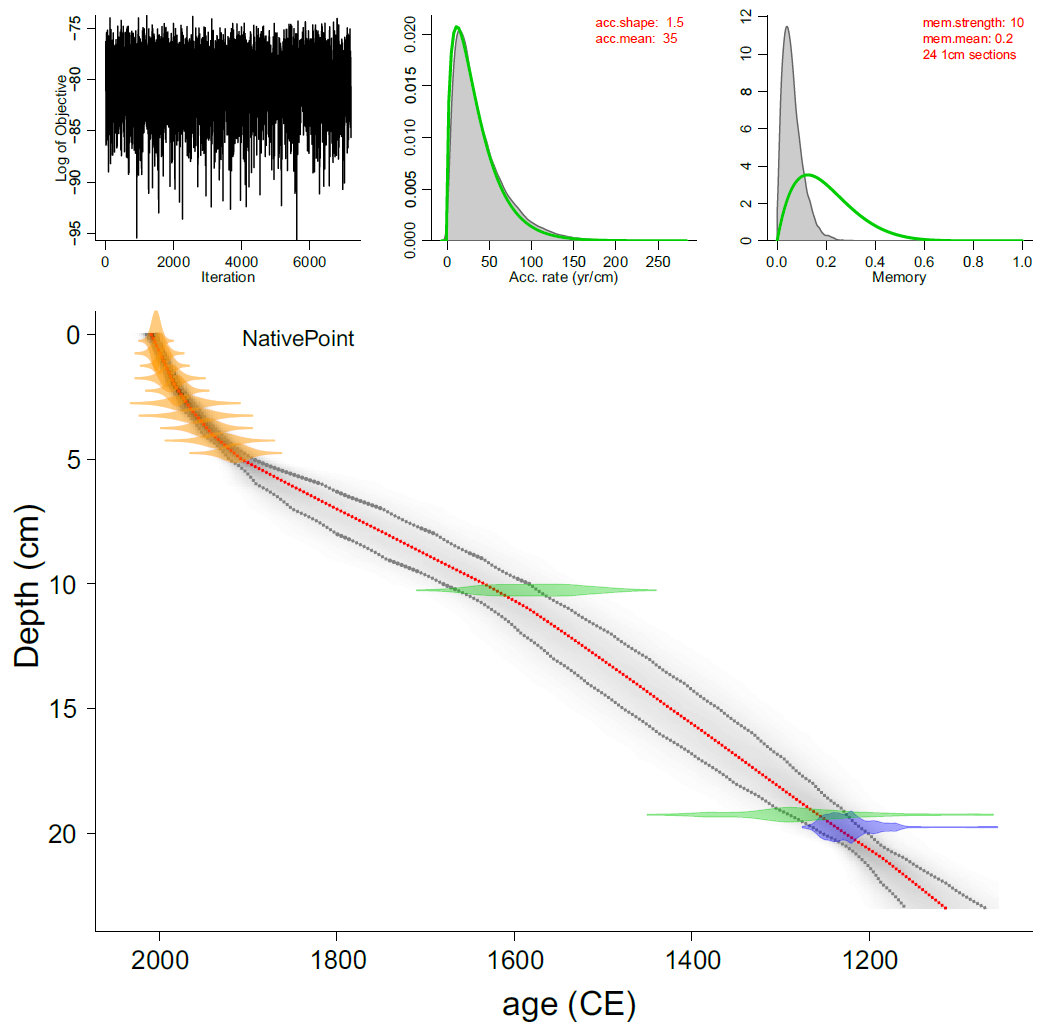


Fig. S1. Bayesian age-depth model from Native Point sediment cores NP-1 and NP-2 using R‑package “bacon v2.5” [^5^](#_ENREF_5).


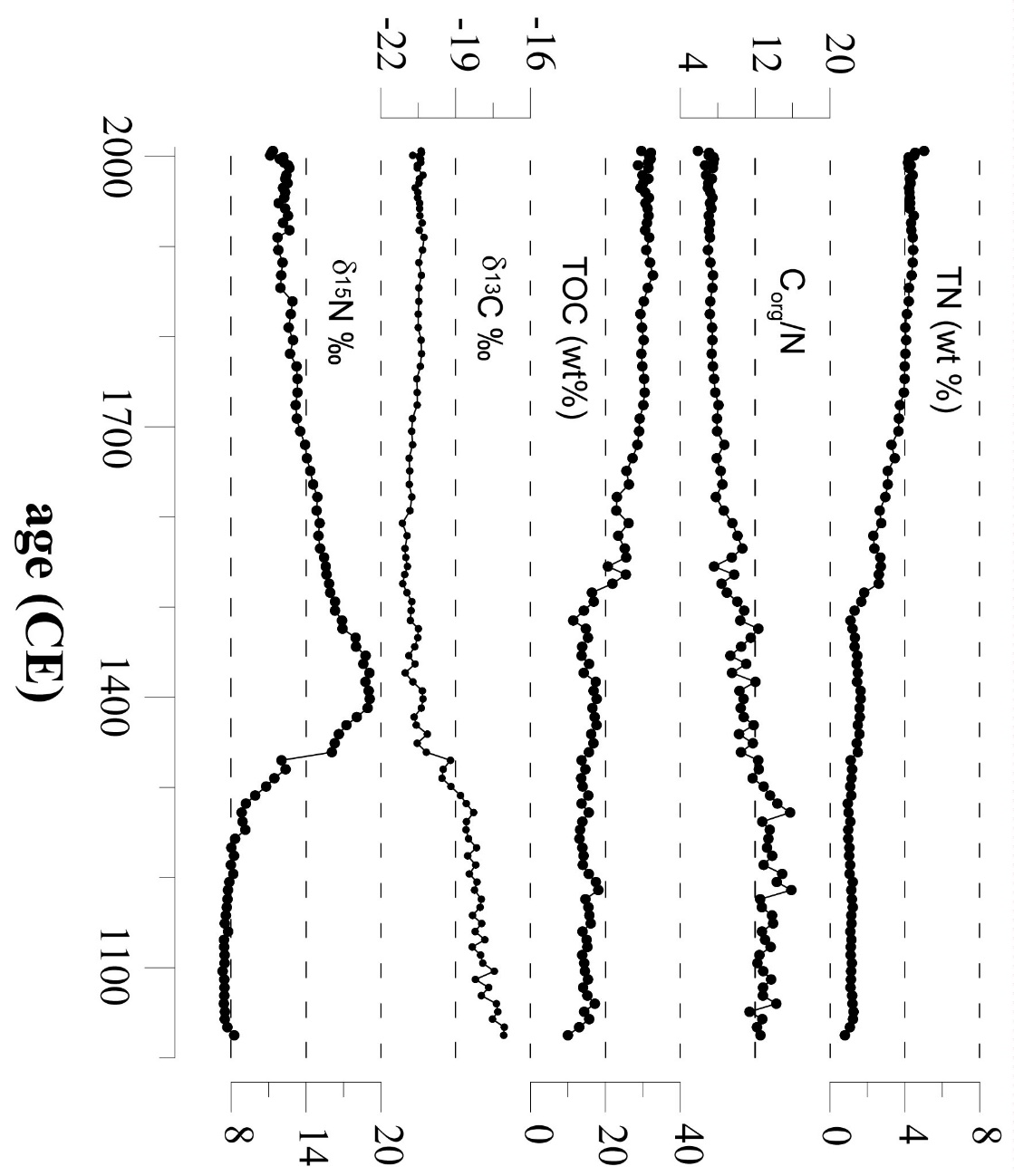


Fig. S2. Results from ^13^C and ^15^N stable isotope measurements from core NP-3.


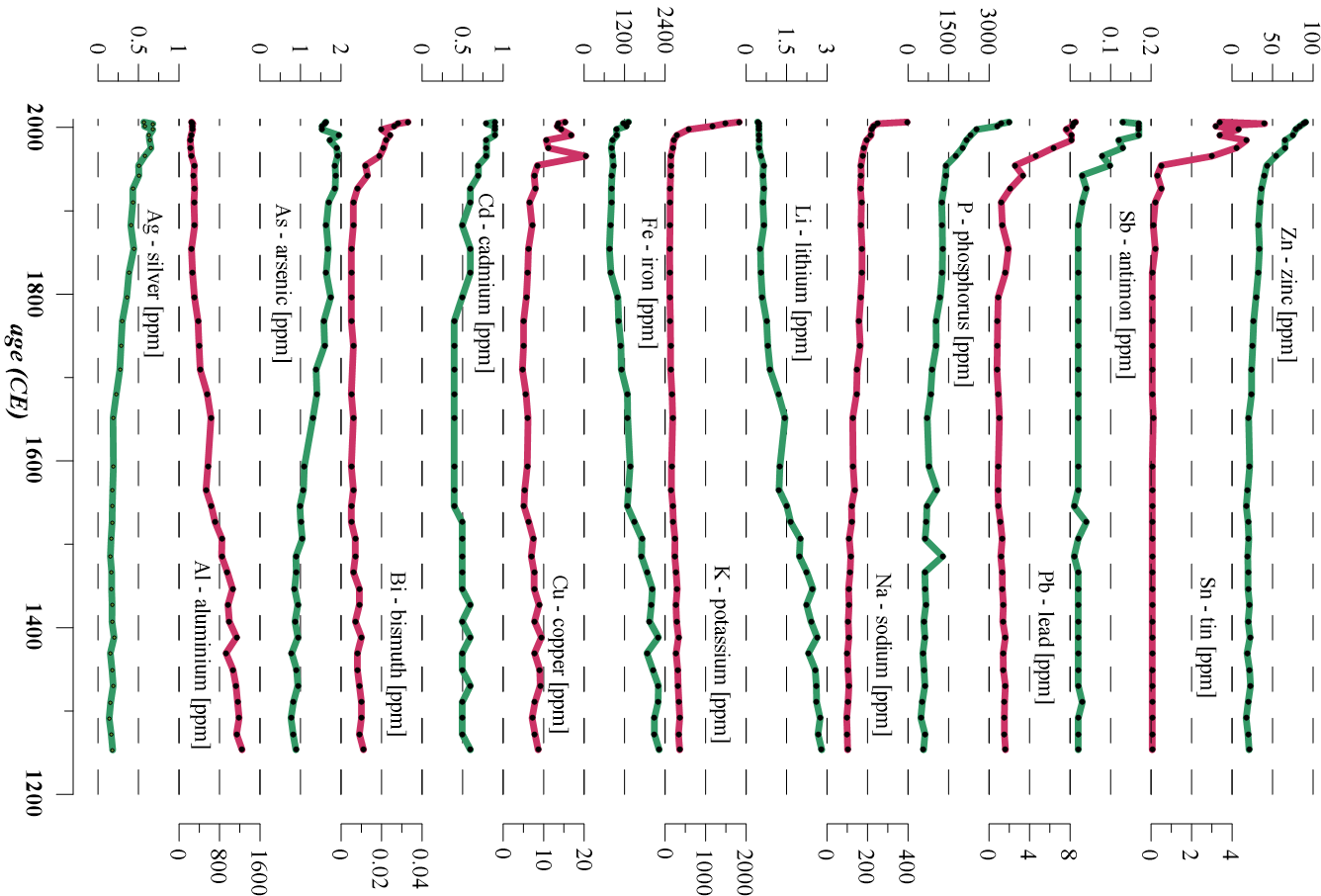


Fig. S3. Concentrations of selected metal in sediment core NP-2.


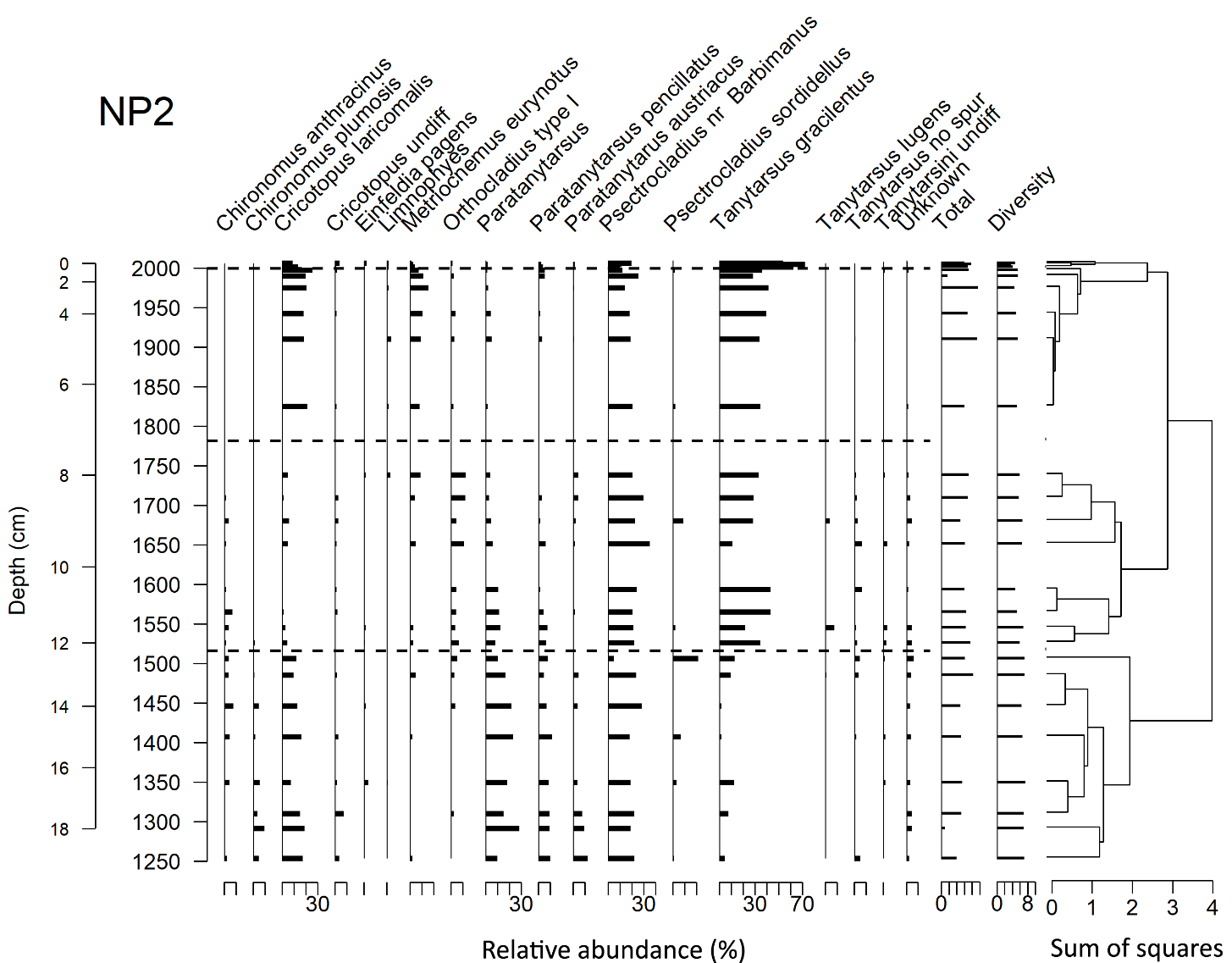


Fig. S4. Relative abundance (%) of chironomid head capsules identified from Native Point sediment core NP-2. Only taxa greater than 2% relative abundance in at least 2 samples are shown. The total sum of head capsules (HC) enumerated per interval are shown; 1.5, 18, and 19 cm are below 50 HC. A constrained cluster dendrogram (Sum of squares) and significant zones as determined by a broken stick model are shown.

**
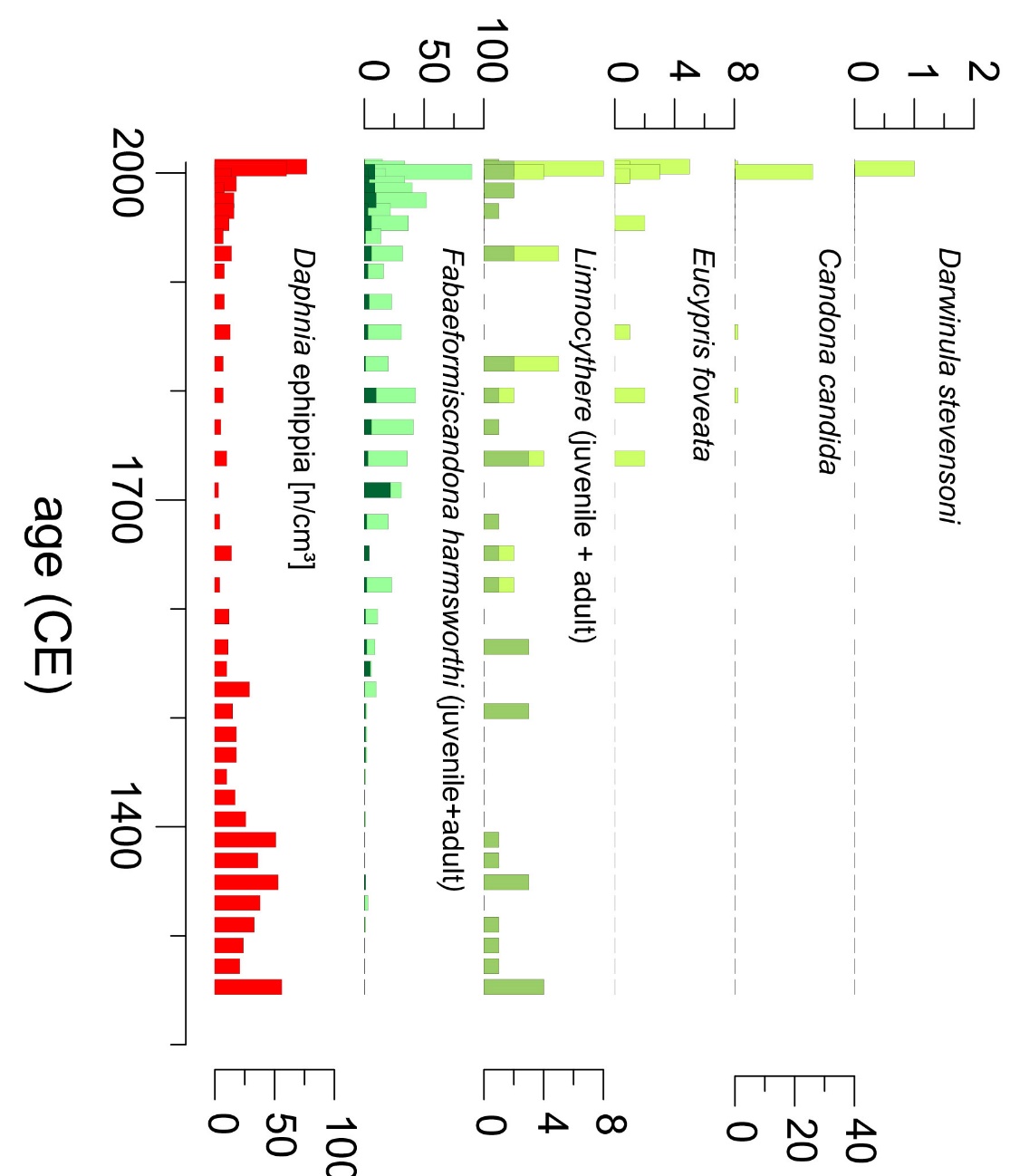
**

Fig. S5. Counting per cm³ of *Daphnia* ephippia and ostracod valves from Native Point sediment core NP-2. Ostracod carapace counted as two individual valves.

Table S1. Overview of water chemistry of selected ponds on Southampton Island. SiteID – site name, Long (N) – longitude north, Lat (W) – latitude west, date – calendar date in 2006, WD – water depth [m], EC – electric conductivity [µS cm^-1^], WT – water temperature [°C], analyses from filtered water samples [mg L^-1^]: NH3-N – ammonium-nitrogen, NO2-N nitrite-nitrogen, TKN-N – total Kjeldahl nitrogen-nitrogen, TP-P – total phosphate-phosphorus, TP-P-UF total phosphate-phosphorus from unfiltered water samples, CHLA-COR Chlorophyll a corrected [µg L^-1^], analyses from unfiltered water samples [mg L^-1^]: DOC – dissolved organic carbon, SIO2 – silicate, DIC – dissolved inorganic carbon, CA – calcium, NA – sodium, MG – magnesium, K – potassium, SO4 – sulfate, CL – chloride; cumulative statistics of the selected ponds excluding Bung Stick Pond (mean, max and min), Mallory et al. 2006 – mean values from past limnological investigations on Southampton Island [^6^](#_ENREF_6).

| SiteID | Long (N) | Lat (W) | date 2006 | WD | pH | EC | WT | NH3-N | NO2-N | TKN-N | TP-P | TP-P-UF | CHLA- COR | DOC | SIO2 | DIC | CA | NA | MG | K | SO4 | CL |
| --- | --- | --- | --- | --- | --- | --- | --- | --- | --- | --- | --- | --- | --- | --- | --- | --- | --- | --- | --- | --- | --- | --- |
| Bung Stick Pond | 63°45.653' | 82°30.569' | 22.07. | 0.6 | 8.9 | 230 | 15.7 | 0.205 | 0.004 | 3.250 | 0.0955 | 0.1300 | 0.1 | 24.7 | 1.33 | 25.2 | 38.8 | 13.00 | 6.20 | 0.31 | 4.14 | 23.60 |
| Lake Datalogger | 64°12.772' | 82°31.176' | 23.07. | 5.5 | 6.6 | 24 | 12.1 | 0.021 | 0.001 | 0.207 | 0.0051 | 0.0037 | 0.4 | 2.5 | 0.23 | 2.8 | 2.5 | 1.64 | 0.56 | 0.29 | 1.05 | 2.13 |
| Lake 091 | 64°12.543' | 83°22.543' | 23.07. | 1.0 | 7.7 | 349 | 18.5 | 0.019 | 0.005 | 0.511 | 0.0062 | 0.0099 | 0.8 | 6.7 | 1.99 | 36.2 | 55.9 | 17.10 | 7.74 | 0.67 | 13.40 | 30.50 |
| Lake 092 | 64°13.335' | 83°23.008' | 23.07. | 0.8 | 7.8 | 100 | 20.1 | 0.016 | 0.004 | 0.265 | 0.0043 | 0.0086 | 1.2 | 3.1 | 0.70 | 13.1 | 20.1 | 1.70 | 1.02 | 0.35 | 3.96 | 2.09 |
| Lake 093 | 64°14.519' | 83°26.250' | 23.07. | 3.6 | 7.6 | 122 | 13.8 | 0.012 | 0.002 | 0.217 | 0.0044 | 0.0074 | 0.4 | 2.9 | 0.37 | 14.2 | 20.7 | 5.19 | 1.81 | 0.49 | 3.22 | 7.77 |
| Lake 101 | 64°11.376' | 83°09.559' | 24.07. | 0.7 | 7.8 | 218 | 14.9 | 0.022 | 0.004 | 0.508 | 0.0047 | 0.0063 | 0.8 | 6.5 | 2.00 | 25.1 | 36.6 | 7.94 | 5.14 | 1.33 | 10.80 | 13.20 |
| Lake 102 | 64°14.184' | 83°04.091' | 24.07. | 0.7 | 7.8 | 163 | 15.2 | 0.015 | 0.006 | 0.179 | 0.0016 | 0.0017 | 0.1 | 1.0 | 1.01 | 22.8 | 30.4 | 3.08 | 4.19 | 0.44 | 2.43 | 4.25 |
| Lake 103 | 64°18.112' | 82°48.551' | 24.07. | 0.5 | 7.7 | 94 | 17.5 | 0.020 | 0.005 | 0.490 | 0.0040 | 0.0075 | 2.7 | 6.3 | 0.35 | 11.9 | 16.0 | 3.78 | 1.16 | 0.87 | 0.38 | 5.74 |
| Lake 104 | 64°16.036' | 82°50.869' | 24.07. | 0.8 | 8.0 | 222 | 17.9 | 0.015 | 0.006 | 0.487 | 0.0048 | 0.0111 | 0.8 | 5.3 | 0.58 | 25.6 | 35.1 | 8.54 | 6.30 | 1.01 | 10.40 | 11.40 |
| Lake 105 | 64°11.834' | 82°51.722' | 24.07. | 0.7 | 8.1 | 437 | 20.9 | 0.071 | 0.005 | 1.340 | 0.0128 | 0.0215 | 1.1 | 13.6 | 1.65 | 32.4 | 57.2 | 38.80 | 10.60 | 2.74 | 20.30 | 82.40 |
|  |  | Mean |  | 1.6 | 7.7 | 192 | 16.8 | 0.023 | 0.004 | 0.467 | 0.0053 | 0.0086 | 0.9 | 5.3 | 0.99 | 20.5 | 30.5 | 9.75 | 4.28 | 0.91 | 7.33 | 17.72 |
|  |  | Max |  | 5.5 | 8.1 | 437 | 20.9 | 0.071 | 0.006 | 1.340 | 0.0128 | 0.0215 | 2.7 | 13.6 | 2.00 | 36.2 | 57.2 | 38.80 | 10.60 | 2.74 | 20.30 | 82.40 |
|  |  | Min |  | 0.5 | 6.6 | 24 | 12.1 | 0.012 | 0.001 | 0.179 | 0.0016 | 0.0017 | 0.1 | 1.0 | 0.23 | 2.8 | 2.5 | 1.64 | 0.56 | 0.29 | 0.38 | 2.09 |
| Mallory et al. 2006 |  |  |  | 2 | 7.9 | NA | NA | NA | NA | 0.716 | NA | NA | NA | NA | NA | NA | NA | NA | NA | NA | NA | NA |

Table S2. Activity of ^210^Po in Core NP-1 Sediment. Sample – sample number, top – sediment sample core depth, bottom – sediment sample core depth, wet.wt – weight of wet sample, dry.wt – weight of dry sample, accu.dry.wt – accumulated weight of dry sample, comp.depth – compressed sediment core depth, uncomp.depth – uncompressed sediment core depth, porosity – porosity, ^210^Po activity –activity of ^210^Po, ^210^Po unsupp. activity – unsupported activity of ^210^Po, age CRS model – ages inferred from Constant Rate of Supply (CRS) model.

| Sample | top  (cm) | bottom  (cm) | wet.wt  (g) | dry.wt  (g) | accu  dry.wt  (g cm^-2^) | comp.  depth  (cm) | uncomp.  depth  (cm) | porosity | ^210^Po activity  (Bq g^-1^) | ^210^Po unsupp. activity  (Bq g^-1^) | age  CRS model |
| --- | --- | --- | --- | --- | --- | --- | --- | --- | --- | --- | --- |
| 1 | 0 | 0.5 | 9.73 | 1.26 | 0.04 | 0.26 | 0.27 | 92.36 | 9.40 | 8.31 | 2004 |
| 2 | 0.5 | 1 | 12.87 | 2.22 | 0.10 | 0.60 | 0.98 | 89.62 | 7.67 | 6.58 | 2000 |
| 3 | 1 | 1.5 | 14.59 | 2.17 | 0.16 | 0.98 | 1.54 | 91.15 |  |  |  |
| 4 | 1.5 | 2 | 16.77 | 3.24 | 0.25 | 1.42 | 2.53 | 88.25 | 5.97 | 4.88 | 1988 |
| 5 | 2 | 2.5 | 9.67 | 2.81 | 0.33 | 1.66 | 4.22 | 81.45 | 4.39 | 3.30 | 1980 |
| 6 | 2.5 | 3 | 20.18 | 5.24 | 0.48 | 2.16 | 5.89 | 83.68 | 3.49 | 2.40 | 1971 |
| 7 | 3 | 3.5 | 20.79 | 5.61 | 0.64 | 2.68 | 7.66 | 82.96 |  |  |  |
| 8 | 3.5 | 4 | 16.45 | 4.73 | 0.77 | 3.09 | 9.49 | 81.68 | 2.29 | 1.20 | 1947 |
| 9 | 4 | 4.5 | 17.13 | 5.28 | 0.92 | 3.51 | 11.54 | 80.15 |  |  |  |
| 10 | 4.5 | 5 | 15.15 | 4.34 | 1.05 | 3.88 | 13.33 | 81.75 | 2.07 | 0.98 | 1914 |
| 11 | 5 | 5.5 | 20.31 | 5.64 | 1.21 | 4.39 | 15.16 | 82.39 |  |  |  |
| 12 | 5.5 | 6 | 19.36 | 5.51 | 1.36 | 4.87 | 17.04 | 81.89 | 1.16 | 0.07 | NA |
| 13 | 6 | 6.5 | 19.58 | 5.50 | 1.52 | 5.35 | 18.89 | 82.16 |  |  |  |
| 14 | 6.5 | 7 | 18.44 | 5.26 | 1.67 | 5.81 | 20.75 | 81.84 | 1.09 | 0.00 | NA |
| 15 | 7 | 7.5 | 18.08 | 5.00 | 1.81 | 6.26 | 22.52 | 82.48 |  |  |  |
| 16 | 7.5 | 8 | 19.65 | 5.51 | 1.97 | 6.75 | 24.36 | 82.20 |  |  |  |
| 17 | 8 | 8.5 | 17.64 | 4.89 | 2.1 | 7.19 | 26.12 | 82.43 |  |  |  |
| 18 | 8.5 | 9 | 18.65 | 5.54 | 2.26 | 7.65 | 28.10 | 80.98 |  |  |  |
| 19 | 9 | 9.5 | 18.63 | 5.24 | 2.41 | 8.11 | 29.93 | 82.13 |  |  |  |
| 20 | 9.5 | 10 | 15.04 | 4.20 | 2.53 | 8.48 | 31.65 | 82.28 |  |  |  |
| 21 | 10 | 11 | 41.04 | 10.60 | 2.83 | 9.51 | 33.83 | 83.78 |  |  |  |
| 22 | 11 | 12 | 30.04 | 7.77 | 3.05 | 10.27 | 35.73 | 83.76 |  |  |  |
| 23 | 12 | 13 | 33.73 | 8.94 | 3.3 | 11.11 | 37.78 | 83.30 |  |  |  |
| 24 | 13 | 14 | 27.10 | 7.33 | 3.51 | 11.79 | 39.72 | 82.91 |  |  |  |
| 25 | 14 | 15 | 26.79 | 7.27 | 3.72 | 12.46 | 41.66 | 82.85 |  |  |  |
| 26 | 15 | 16 | 27.03 | 7.23 | 3.92 | 13.13 | 43.57 | 83.13 |  |  |  |
| 27 | 16 | 17 | 31.99 | 9.43 | 4.19 | 13.92 | 45.85 | 81.15 |  |  |  |
| 28 | 17 | 18 | 31.34 | 9.37 | 4.46 | 14.69 | 48.16 | 80.84 |  |  |  |
| 29 | 18 | 19 | 37.02 | 12.19 | 4.80 | 15.59 | 50.89 | 78.56 |  |  |  |
| 30 | 19 | 20 | 35.63 | 10.83 | 5.11 | 16.46 | 53.35 | 80.47 |  |  |  |
| 31 | 20 | 21 | 36.77 | 12.10 | 5.45 | 17.35 | 56.07 | 78.58 |  |  |  |
| 32 | 21 | 22 | 26.68 | 8.73 | 5.70 | 18.00 | 58.53 | 78.72 |  |  |  |
| 33 | 22 | 23 | 24.95 | 8.86 | 5.95 | 18.6 | 61.23 | 76.56 |  |  |  |

**Table S3.** AMS radiocarbon dates and δ^13^C from Native Point core NP-2. The sample ages were measured after acid extraction (HCl) at the AMS Cologne facility, in Cologne, Germany (COL) or Keck Carbon Cycle AMS Facility (UCIAMS). Laboratory identifier (Lab Code), sample depth, materials chosen as well as radiocarbon ages and calendar ages are given. F^14^C –fraction of modern and error. The radiocarbon ages of all samples were calibrated into calendar years (CE) using OxCal v. 4.3 [^7^](#_ENREF_7) and applying the INTCAL13 calibration curve [^8^](#_ENREF_8). cmblf = centimeters below lake floor. Local marine reservoir ages of ΔR = 263 ± 48 years [^9^](#_ENREF_9).

| **lab code** | **sample depth**  **(cmblf)** | **material** | **F^14^C** | **F^14^C error** | **δ^13^C (‰)** | **^14^C age**  **(yrs BP)** | **error**  **(yrs)** | **ΔR** | **calendar age (CE) range of 95.4% [median probability]** |
| --- | --- | --- | --- | --- | --- | --- | --- | --- | --- |
| **UCIAMS-168600/**  **ULA-5743** | 10.00-10.50 | organic | 0.8751 | 0.0016 | -23.1 | 1,070 | 15 | 263 ± 48 | 1435 – 1635 [1509] |
| **COL4234** | 19.00-19.50 | organic | 0.8352 | 0.0036 | -20.9 | 1,447 | 35 | 263 ± 48 | 1075 – 1311 [1218] |
| **UCIAMS-168601/**  **ULA-5744** | 19.50-19.75 | terrestrial  plant | 0.9029 | 0.0017 | -22.8 | 820 | 20 | NA | 1181 – 1263 [1228] |

**Table S4.** Overview of metal analysis results (ppm dry wt) in sediment core NP-2. Ag - Silver, Al - Aluminium, As - Arsenic, Bi - Bismuth, Cd - Cadmium, Cu – Copper, Fe - Iron, K - Potassium, Li - Lithium, Na - Sodium, P - Phosphorus, Pb - Lead, Sb - Antimony, Sn - Tin, Zn - Zinc

| **ID** | **Top** | **Bottom** | **Age** | **Ag** | **Al** | **As** | **Bi** | **Cd** | **Cu** | **Fe** | **K** | **Li** | **Na** | **P** | **Pb** | **Sb** | **Sn** | **Zn** |
| --- | --- | --- | --- | --- | --- | --- | --- | --- | --- | --- | --- | --- | --- | --- | --- | --- | --- | --- |
| 1 | 0 | 0.25 | 2006 | 0.571 | 254 | 1.62 | 0.033 | 0.9 | 15.2 | 1300 | 1820 | 0.43 | 396 | 3740 | 8.56 | 0.13 | 3.4 | 91.3 |
| 2 | 0.25 | 0.5 | 2004 | 0.683 | 278 | 1.59 | 0.028 | 0.8 | 13.6 | 1140 | 1480 | 0.47 | 249 | 3440 | 8.31 | 0.17 | 5.6 | 88.7 |
| 3 | 0.5 | 1.0 | 2001 | 0.580 | 259 | 1.53 | 0.026 | 0.9 | 13.3 | 1230 | 1150 | 0.47 | 229 | 3290 | 8.22 | 0.17 | 3.2 | 84.3 |
| 4 | 1.0 | 1.5 | 1997 | 0.683 | 275 | 1.53 | 0.020 | 0.9 | 14.3 | 944 | 567 | 0.47 | 220 | 2540 | 7.62 | 0.17 | 4.3 | 79.2 |
| 5 | 1.5 | 2.0 | 1990 | 0.637 | 242 | 1.95 | 0.024 | 0.9 | 16.8 | 962 | 279 | 0.48 | 219 | 2330 | 8.16 | 0.17 | 3.4 | 75.5 |
| 6 | 2.0 | 2.5 | 1984 | 0.642 | 223 | 1.72 | 0.022 | 0.8 | 10.6 | 834 | 200 | 0.49 | 195 | 2160 | 8.14 | 0.12 | 4.7 | 66.1 |
| 7 | 2.5 | 3.0 | 1975 | 0.662 | 237 | 1.89 | 0.021 | 0.8 | 11.1 | 793 | 180 | 0.48 | 184 | 2010 | 6.37 | 0.13 | 4.2 | 65.9 |
| 8 | 3.0 | 3.5 | 1965 | 0.580 | 253 | 1.91 | 0.019 | 0.8 | 20.3 | 830 | 133 | 0.54 | 176 | 1760 | 4.67 | 0.08 | 3.0 | 54.4 |
| 9 | 3.5 | 4.0 | 1954 | 0.507 | 308 | 1.85 | 0.012 | 0.7 | 8.4 | 847 | 125 | 0.64 | 169 | 1420 | 2.62 | 0.10 | 0.50 | 43.4 |
| 10 | 4.0 | 4.5 | 1942 | 0.510 | 297 | 1.86 | 0.013 | 0.7 | 7.6 | 815 | 125 | 0.62 | 170 | 1390 | 3.34 | 0.03 | 0.30 | 40.6 |
| 11 | 4.5 | 5.0 | 1926 | 0.436 | 314 | 1.85 | 0.008 | 0.6 | 7.9 | 795 | 116 | 0.65 | 166 | 1330 | 2.11 | 0.04 | 0.50 | 36.9 |
| 12 | 5.0 | 5.5 | 1910 | 0.436 | 298 | 1.69 | 0.006 | 0.6 | 6.5 | 798 | 115 | 0.62 | 171 | 1250 | 1.23 | 0.03 | 0.20 | 35.1 |
| 13 | 5.5 | 6.0 | 1882 | 0.411 | 314 | 1.63 | 0.006 | 0.5 | 7.1 | 764 | 113 | 0.66 | 169 | 1270 | 1.34 | 0.02 | 0.10 | 33.3 |
| 14 | 6.0 | 6.5 | 1854 | 0.448 | 251 | 1.68 | 0.005 | 0.6 | 6.2 | 736 | 104 | 0.53 | 174 | 1280 | 1.94 | 0.02 | 0.20 | 34.5 |
| 15 | 6.5 | 7.0 | 1825 | 0.387 | 271 | 1.63 | 0.005 | 0.6 | 6.0 | 781 | 106 | 0.55 | 174 | 1270 | 1.64 | 0.02 | 0.05 | 32.9 |
| 16 | 7.0 | 7.5 | 1796 | 0.360 | 308 | 1.74 | 0.005 | 0.5 | 5.7 | 989 | 108 | 0.60 | 167 | 1200 | 0.92 | 0.02 | 0.05 | 30.2 |
| 17 | 7.5 | 8.0 | 1767 | 0.306 | 380 | 1.57 | 0.005 | 0.4 | 5.0 | 1010 | 120 | 0.76 | 159 | 1040 | 0.87 | 0.02 | 0.05 | 27.3 |
| 18 | 8.0 | 8.5 | 1738 | 0.290 | 399 | 1.59 | 0.006 | 0.4 | 5.0 | 1080 | 125 | 0.80 | 165 | 1050 | 0.84 | 0.02 | 0.05 | 26.1 |
| 19 | 8.5 | 9.0 | 1709 | 0.275 | 422 | 1.38 | <0.005 | 0.4 | 4.7 | 1090 | 127 | 0.89 | 150 | 896 | 0.80 | 0.02 | 0.05 | 24.8 |
| 20 | 9.0 | 9.5 | 1680 | 0.232 | 560 | 1.40 | 0.005 | 0.4 | 5.6 | 1270 | 160 | 1.19 | 147 | 841 | 0.94 | 0.02 | 0.05 | 24.8 |
| 21 | 9.5 | 10.0 | 1651 | 0.192 | 636 | 1.30 | 0.006 | 0.4 | 6.1 | 1280 | 178 | 1.44 | 128 | 717 | 1.05 | 0.02 | 0.10 | 21.1 |
| 22 | 10.5 | 11.0 | 1593 | 0.196 | 573 | 1.09 | 0.005 | 0.4 | 5.9 | 1350 | 148 | 1.25 | 130 | 777 | 0.93 | 0.02 | 0.05 | 22.3 |
| 23 | 11.0 | 11.5 | 1565 | 0.187 | 537 | 1.07 | 0.006 | 0.4 | 5.3 | 1300 | 146 | 1.22 | 137 | 1070 | 0.90 | 0.02 | 0.05 | 19.9 |
| 24 | 11.5 | 12.0 | 1545 | 0.182 | 644 | 0.98 | 0.005 | 0.4 | 5.1 | 1270 | 180 | 1.51 | 122 | 702 | 0.95 | 0.01 | 0.05 | 18.1 |
| 25 | 12.0 | 12.5 | 1526 | 0.180 | 716 | 1.00 | 0.005 | 0.5 | 6.3 | 1480 | 195 | 1.65 | 123 | 689 | 1.15 | 0.04 | 0.05 | 20.3 |
| 26 | 12.5 | 13.0 | 1506 | 0.167 | 852 | 1.04 | 0.007 | 0.5 | 7.4 | 1700 | 235 | 2.00 | 111 | 636 | 1.32 | 0.02 | 0.05 | 21.3 |
| 27 | 13.0 | 13.5 | 1485 | 0.159 | 846 | 0.88 | 0.007 | 0.5 | 6.9 | 1690 | 229 | 1.97 | 118 | 1280 | 1.18 | 0.01 | 0.05 | 20.0 |
| 28 | 13.5 | 14.0 | 1466 | 0.175 | 959 | 0.89 | 0.006 | 0.5 | 7.6 | 1850 | 263 | 2.25 | 115 | 629 | 1.33 | 0.02 | 0.05 | 20.3 |
| 29 | 14.0 | 14.5 | 1446 | 0.174 | 1060 | 0.85 | 0.009 | 0.5 | 7.6 | 2010 | 291 | 2.47 | 107 | 640 | 1.34 | 0.02 | 0.05 | 20.2 |
| 30 | 14.5 | 15.0 | 1427 | 0.180 | 972 | 0.93 | 0.009 | 0.6 | 8.8 | 1980 | 258 | 2.24 | 110 | 667 | 1.44 | 0.02 | 0.05 | 22.2 |
| 31 | 15.0 | 15.5 | 1407 | 0.178 | 997 | 0.86 | 0.007 | 0.5 | 7.7 | 1930 | 272 | 2.40 | 106 | 587 | 1.37 | 0.02 | 0.05 | 20.3 |
| 32 | 15.5 | 16.0 | 1388 | 0.204 | 1140 | 0.93 | 0.010 | 0.6 | 9.4 | 2190 | 319 | 2.63 | 109 | 627 | 1.63 | 0.02 | 0.05 | 22.8 |
| 33 | 16.0 | 16.5 | 1369 | 0.158 | 933 | 0.78 | 0.008 | 0.5 | 7.7 | 1850 | 263 | 2.31 | 102 | 552 | 1.41 | 0.02 | 0.05 | 19.1 |
| 34 | 16.5 | 17.0 | 1349 | 0.179 | 1070 | 0.90 | 0.008 | 0.5 | 9.0 | 2020 | 300 | 2.56 | 105 | 596 | 1.43 | 0.02 | 0.05 | 22.2 |
| 35 | 17.0 | 17.5 | 1330 | 0.194 | 1130 | 0.93 | 0.009 | 0.6 | 9.1 | 2190 | 312 | 2.60 | 109 | 629 | 1.63 | 0.02 | 0.05 | 23.4 |
| 36 | 17.5 | 18.0 | 1310 | 0.162 | 1160 | 0.82 | 0.010 | 0.5 | 7.8 | 2180 | 333 | 2.61 | 105 | 534 | 1.56 | 0.03 | 0.05 | 20.4 |
| 37 | 18.0 | 18.5 | 1291 | 0.144 | 1190 | 0.76 | 0.010 | 0.5 | 7.1 | 2070 | 351 | 2.74 | 98 | 478 | 1.53 | 0.02 | 0.05 | 18.0 |
| 38 | 18.5 | 19.0 | 1272 | 0.168 | 1140 | 0.81 | 0.009 | 0.5 | 7.8 | 2070 | 319 | 2.66 | 101 | 632 | 1.54 | 0.02 | 0.05 | 20.2 |
| 39 | 19.0 | 19.5 | 1253 | 0.187 | 1250 | 0.88 | 0.011 | 0.6 | 8.6 | 2210 | 349 | 2.80 | 103 | 579 | 1.64 | 0.02 | 0.05 | 21.6 |

**Table S5.** Overview of metal Enrichment Factors (EF) in relation to pre-industrial era. Ag - Silver, Al - Aluminium, As - Arsenic, Bi - Bismuth, Cd - Cadmium, Cu – Copper, Fe - Iron, K - Potassium, Li - Lithium, Na - Sodium, P - Phosphorus, Pb - Lead, Sb - Antimony, Sn - Tin, Zn - Zinc

| **era** | **Ag** | **Al** | **As** | **Bi** | **Cd** | **Cu** | **Fe** | **K** | **Li** | **Na** | **P** | **Pb** | **Sb** | **Sn** | **Zn** |
| --- | --- | --- | --- | --- | --- | --- | --- | --- | --- | --- | --- | --- | --- | --- | --- |
| post-1950/pre-1800 | 2.9 | 0.3 | 1.6 | 3.1 | 1.7 | 1.9 | 0.6 | 2.7 | 0.3 | 1.9 | 3.1 | 4.6 | 10.0 | 21.1 | 3.0 |
| post-1950/pre-1300 | 3.7 | 0.2 | 2.1 | 2.3 | 1.6 | 1.8 | 0.5 | 1.9 | 0.2 | 2.2 | 4.5 | 4.4 | 11.8 | 71.8 | 3.6 |

**SI References**

1 Environment Canada. Manual of analytical methods. *National Laboratory for Environmental Testing, Canadian Centre for Inland Waters, Burlington, Ontario, Canada* (1994).

2 Werner, R. A., Bruch, B. A. & Brand, W. A. ConFlo III – an interface for high precision δ13C and δ15N analysis with an extended dynamic range. *Rapid Communications in Mass Spectrometry* **13**, 1237-1241, doi:10.1002/(SICI)1097-0231(19990715)13:13<1237::AID-RCM633>3.0.CO;2-C (1999).

3 Roberts, S. *et al.* Mercury and metal(loid) deposition to remote Nova Scotia lakes from both local and distant sources. *Sci. Total Environ.* **675**, 192-202, doi:https://doi.org/10.1016/j.scitotenv.2019.04.167 (2019).

4 Wiklund, J. A. *et al.* Widespread Atmospheric Tellurium Contamination in Industrial and Remote Regions of Canada. *Environ. Sci. Technol.* **52**, 6137-6145, doi:10.1021/acs.est.7b06242 (2018).

5 Blaauw, M. & Christen, J. A. Flexible paleoclimate age-depth models using an autoregressive gamma process. *Bayesian Analysis*, 457-474, doi:10.1214/ba/1339616472 (2011).

6 Mallory, M. L., Fontaine, A. J., Smith, P. A., Wiebe Robertson, M. O. & Gilchrist, H. G. Water chemistry of ponds on Southampton Island, Nunavut, Canada: effects of habitat and ornithogenic inputs. *Arch. Hydrobiol.* **166**, 411-432, doi:10.1127/0003-9136/2006/0166-0411 (2006).

7 Bronk Ramsey, C. Bayesian Analysis of Radiocarbon Dates. *Radiocarbon* **51**, 337-360, doi:10.1017/s0033822200033865 (2009).

8 Reimer, P. J. *et al.* IntCal13 and Marine13 radiocarbon age calibration curves 0-50,000 years cal BP. *Radiocarbon* **55**, 1869-1887 (2013).

9 Ross, M., Utting, D. J., Lajeunesse, P. & Kosar, K. G. A. Early Holocene deglaciation of northern Hudson Bay and Foxe Channel constrained by new radiocarbon ages and marine reservoir correction. *Quatern. Res.* **78**, 82-94, doi:10.1016/j.yqres.2012.03.001 (2012).
